# Intraoperative Perfusion Assessment in Enhanced Reality Using Quantitative Optical Imaging: An Experimental Study in a Pancreatic Partial Ischemia Model †

**DOI:** 10.3390/diagnostics11010093

**Published:** 2021-01-08

**Authors:** Taiga Wakabayashi, Manuel Barberio, Takeshi Urade, Raoul Pop, Emilie Seyller, Margherita Pizzicannella, Pietro Mascagni, Anne-Laure Charles, Yuta Abe, Bernard Geny, Andrea Baiocchini, Yuko Kitagawa, Jacques Marescaux, Eric Felli, Michele Diana

**Affiliations:** 1 Research Institute against Digestive Cancer (IRCAD), 67000 Strasbourg, France; taiga.wakabayashi@me.com (T.W.); emily.seyller@gmail.com (E.S.); marghe.pizzicannella@gmail.com (M.P.); jacques.marescaux@ircad.fr (J.M.); 2 Department of Surgery, Keio University School of Medicine, Tokyo 160-8582, Japan; abey3666@gmail.com (Y.A.); kitagawa.a3@keio.jp (Y.K.); 3 Institut Hospitalo-Universitaire (IHU), Institute for Minimally Invasive Hybrid Image-Guided Surgery, University of Strasbourg, 67091 Strasbourg, France; manuel.barberio@ihu-strasbourg.eu (M.B.); uradet1125@gmail.com (T.U.); pop.raoul@gmail.com (R.P.); pietro.mascagni@ihu-strasbourg.eu (P.M.); eric.felli@ihu-strasbourg.eu (E.F.); 4 Institute of Physiology, UR3072 ‘Mitochondria, Oxidative Stress and Muscle Protection’, Translational Medicine Federation, Faculty of Medicine, University of Strasbourg, 67000 Strasbourg, France; anne.laure.charles@unistra.fr (A.-L.C.); bernard.geny@chru-strasbourg.fr (B.G.); 5 Department of Visceral, Transplant, Thoracic and Vascular Surgery, University Hospital of Leipzig, 04103 Leipzig, Germany; 6 Department of Surgical Pathology, San Camillo Hospital, 00152 Rome, Italy; baiocchiniandrea@gmail.com; 7 General, Digestive and Endocrine Surgery, Nouvel Hôpital Civil, University of Strasbourg, 67000 Strasbourg, France; 8 ICube Lab, Photonics for Health, University of Strasbourg, 67000 Strasbourg, France

**Keywords:** optical imaging, fluorescence imaging, hyperspectral imaging, pancreatectomy

## Abstract

To reduce the risk of pancreatic fistula after pancreatectomy, a satisfactory blood flow at the pancreatic stump is considered crucial. Our group has developed and validated a real-time computational imaging analysis of tissue perfusion, using fluorescence imaging, the fluorescence-based enhanced reality (FLER). Hyperspectral imaging (HSI) is another emerging technology, which provides tissue-specific spectral signatures, allowing for perfusion quantification. Both imaging modalities were employed to estimate perfusion in a porcine model of partial pancreatic ischemia. Perfusion quantification was assessed using the metrics of both imaging modalities (slope of the time to reach maximum fluorescence intensity and tissue oxygen saturation (StO2), for FLER and HSI, respectively). We found that the HSI-StO2 and the FLER slope were statistically correlated using the Spearman analysis (R = 0.697; *p* = 0.013). Local capillary lactate values were statistically correlated to the HSI-StO2 and to the FLER slope (R = −0.88; *p* < 0.001 and R = −0.608; *p* = 0.0074). HSI-based and FLER-based lactate prediction models had statistically similar predictive abilities (*p* = 0.112). Both modalities are promising to assess real-time pancreatic perfusion. Clinical translation in human pancreatic surgery is currently underway.

## 1. Introduction

Given the technical complexity of pancreatic surgery, the complication rate following pancreatectomy is high (approximately 50% of patients) [1] and this may affect the long-term oncological prognosis negatively [2]. Pancreatic fistula (PF), which represents the leakage of pancreatic fluid into the abdominal cavity, is the most frequent and dreadful adverse event after pancreatectomy [3]. PF can potentially lead to severe conditions such as multiple organ failure, sudden and extensive bleeding and death. The etiology of PF is multifactorial and the existing literature has focused mainly on mechanical causes, such as pancreatic tissue texture, pancreatic duct diameter [4] or type of anastomotic technique used [5]. However, as already pointed out by Strasberg et al., a satisfactory blood flow at the pancreatic stump is necessary to promote proper healing and reduce the risk of PF [6].

Clinical parameters, such as tissue color or the presence of a local pulse, are unreliable to assess the viability of visceral organs intraoperatively [7]. Fluorescence angiography (FA), an intraoperative imaging modality based on the use of near-infrared cameras and on the injection of an exogenous fluorophore (e.g., indocyanine green (ICG)) has shown promising results as a tool to assess the viability of the gastrointestinal tract. The perfusion evaluation is based on the principle that ICG propagates in vital regions but not in ischemic ones. However, the dye diffuses into ischemic regions over time and this causes potential signal misinterpretations. For this reason, an objective quantitative FA method, known as FLER (fluorescence-based enhanced reality), has been introduced and validated [8,9]. For each pixel, FLER computes the speed required by the dye to reach its maximal intensity, creating a perfusion heat map, as explained in detail elsewhere [8,9]. The slope of the time-to-peak obtained by FLER has been previously validated to quantify intestinal ischemia, using reliable biomarkers, such as local capillary lactates and mitochondrial respiration [8,9]. However, the need for the injection of a fluorophore represents the main drawback of FA and sometimes limits its routine use [10,11].

Hyperspectral imaging (HSI) is a relatively new imaging technology applied in medicine [12,13,14,15,16], resulting from the combination of digital photography with spectroscopy and allowing to overcome the limitations of the human eye, which can detect light exclusively within the visual range of the electromagnetic spectrum (400–700 nm). In fact, HSI is able to act within a much broader spectral range, varying according to each hyperspectral imager. HSI provides an objective evaluation of intrinsic biochemical tissue characteristics resulting from tissue-light interactions (reflectance, adsorption and scattering), which generate tissue-specific spectral signatures. HSI has shown promising results in quantifying intestinal perfusion in terms of tissue oxygen saturation (StO_2_) during surgical procedures [17,18]. However, HSI produces static images, and this represents a consistent limitation for its intraoperative use. Consequently, HYPER (hyperspectral-based enhanced reality) has been introduced. HYPER results from the superimposing of the static HSI-based perfusion heat map onto the real-time video of the surgical scene, thereby allowing for HSI-guided intraoperative navigation. The accuracy of HYPER has been recently tested in the porcine bowel ischemia model [19] and to guide anatomical liver resections [20], using similar experimental protocols as the ones employed to validate FLER. Additionally, this technology has proven to be more precise than FLER to recognize marginally perfused gastrointestinal tract areas [21].

Recently, confocal laser endomicroscopy (CLE), a probe-based optical imaging technology has been successfully employed to assess intestinal [22,23] and gastric ischemia [24,25]. However, differently from FLER and HYPER, CLE provides only limited and punctual spatial information.

The aim of this study is to investigate the accuracy of the output measures generated by FLER and HYPER, namely the slope of the time-to-peak and StO_2,_ for the real-time estimation of pancreatic blood perfusion in a porcine pancreatic partial ischemia model. The performance of those two optical imaging technologies is evaluated using reliable ischemia surrogates (local capillary lactates, mitochondrial respiration and histopathological damage) as well as CLE.

## 2. Materials and Methods

### 2.1. Animal Characteristics

The present experimental study is part of the Endoscopic Luminescent Imaging for Oncology Surgery (ELIOS) project, which received full approval from the local Ethical Committee on Animal Experimentation (ICOMETH No. 38.2016.01.085) and by the French Ministry of Superior Education and Research (MESR) under the reference APAFIS #8721-201701301 0316298 v2 on 2 March 2017. All animals used in the experimental laboratory were managed according to French laws for animal use and care, according to the directives of the European Community Council (2010/63/EU) and to ARRIVE guidelines [26].

A total of six male Large White breed pigs (mean weight 47.5 ± 5.2 kg) were involved in this acute study. The animals were made to fast for 24 h with free access to water before surgery. Animals were premedicated, 10 min before surgery, by means of an intramuscular injection of ketamine (20 mg/kg) and azaperone (2 mg/kg). Intravenous propofol (3 mg/kg) was used in combination with rocuronium (0.8 mg/kg) used for induction. Anesthesia was maintained with 2% isoflurane. At the end of the procedures, the pigs were sacrificed with an intravenous injection of a lethal dose of potassium chloride.

### 2.2. Endovascular Pancreatic Embolization and Surgical Procedure

The experimental procedures were performed in a preclinical hybrid operative room. Under general anesthesia and in compliance with surgical aseptic conditions, a 5 French sheath was introduced into the right femoral artery under ultrasound guidance. A 5 French Cobra-2 catheter (Terumo Europe NV, Leuven, Belgium) was placed successively at the origin of the coeliac trunk and the superior mesenteric artery and selective digital subtraction angiography was performed using 10 mL of contrast medium at a rate of 4 mL/s (VISIPAQUE 270, GE Healthcare, Amersham Place, UK).

**Figure 1:**
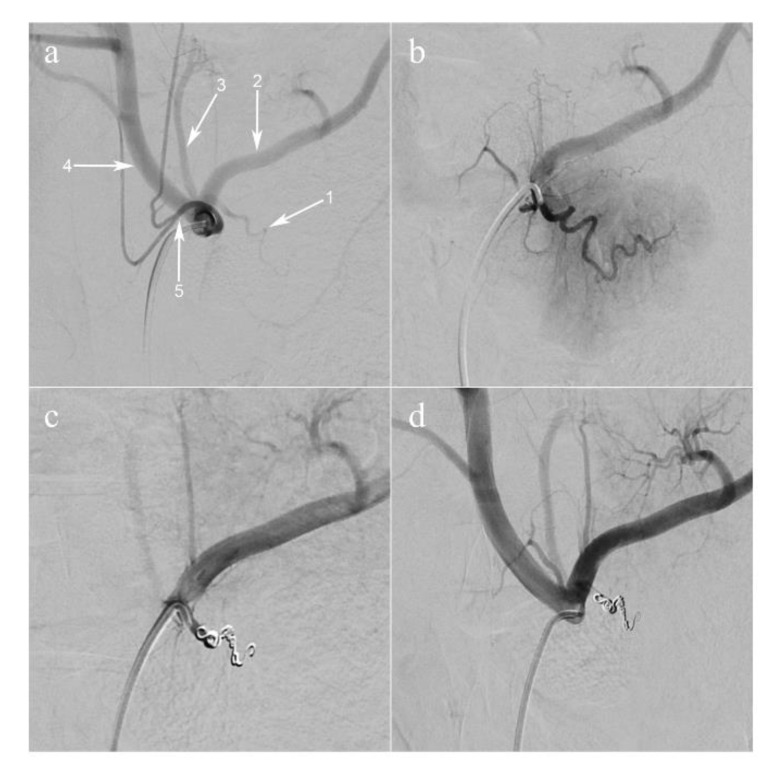
Arterial coil embolization of pancreatic artery. (**a**) Selective injection of the celiac trunk (digital subtraction angiography, anteroposterior view). The dorsal pancreatic artery (1) is shown, branching off the proximal splenic artery (2); (3) the left gastric artery; (4) the common hepatic artery; (5) the common trunk of the left and right phrenic arteries. (**b**) Super-selective injection of the dorsal pancreatic artery. The parenchyma of the pancreatic tail is visualized. (**c**) The dorsal pancreatic artery is occluded with coils. (**d**) Selective injection of the celiac trunk post-embolization.

**Figure 2:**
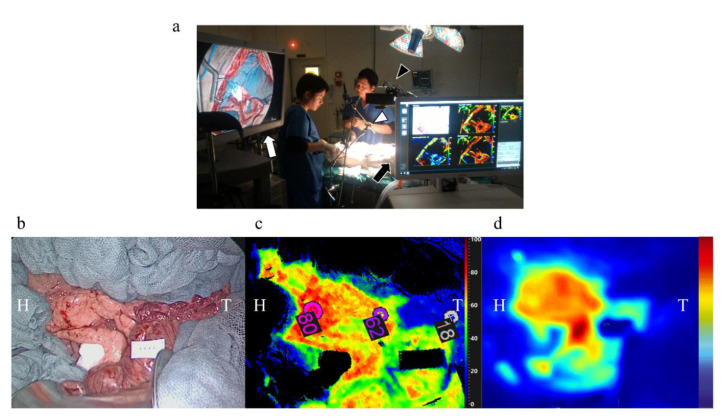
Image acquisition of the pancreas. (**a**) The monitor of the fluorescence-guided enhanced reality system is placed on the lower left side of the operating table (white arrow) and the 30-degree laparoscopic camera is fixed at an articulated arm and set at an approximate distance of 15 cm from the operating field (white arrowhead). The cart of the HSI system is placed on the upper left side of the operating table (black arrow) and the HSI camera is placed approximately at a 30 cm fixed distance from the target (black arrowhead). (**b**) An image under white light, which shows a clear macroscopic sign of pancreatic segmental ischemia in the tail of the pancreas, (**c**) an image elaborated by means of the HSI camera, (**d**) and an image elaborated by means of FLER (**b**–**d**) were obtained in the pancreas of the same pig. H: head of the pancreas, T: tail of the pancreas.

The main pancreatic branch of the splenic artery (equivalent to the dorsal pancreatic artery in humans) was selectively catheterized using a 2.8 French microcatheter (PROGREAT^®^, Terumo Europe NV, Leuven, Belgium). The artery was occluded using large volume 0.020-inch diameter coils (Ruby^®^ Coils, Penumbra Inc., Alameda, CA USA), in order to obtain a selective ischemia of the pancreatic tail and part of the body ( Figure 1; Figure 2b).

At the end of the procedure, the catheters were withdrawn, and hemostasis was achieved by means of manual compression of the femoral artery access site.

Immediately after embolization, a median laparotomy was performed and the pancreas was exposed, taking care to prevent injuries to the pancreatic capsule and vasculature.

### 2.3. Quantitative Perfusion Assessment Steps

A near-infrared (NIR) laparoscopic camera (D-light P™, Karl Storz, Tuttlingen, Germany) and a push-broom CMOS HSI system (TIVITA™ Tissue, Diaspective Vision GmbH, Am Salzhaff, Germany; spatial resolution of 640 × 480 pixels, spectral range from 500 to 1000 nm and 5 nm spectral resolution) were placed on the surgical field **(**Figure 2a). The HSI system provides pseudo-color images containing the perfusion quantification information in terms of tissue oxygen saturation (StO_2_) as immediate output (Figure 2c).

Two hours after completion of the endovascular procedure an HSI acquisition (acquisition time of approximately 8 s) was performed. Successively, a bolus of 0.2 mg/kg ICG (Infracyanine™, Serb laboratories, Paris, France) was administered intravenously and using the NIR laparoscope, FLER was performed (acquisition time of approximately 40 s) (Figure 2d). Based on the perfusion cartography provided via HSI, an investigator (M.B.) manually annotated three regions of interest (ROIs; vital, transition and ischemic zones)**.** As visible in Figure 2, the vital and the ischemic regions were easily recognized, the transition zone was set in between the previous two regions. The StO_2_ quantification pseudo-color image with the three ROIs was superimposed onto the perfusion cartography provided with FLER, and both images were overlaid onto the live video of the surgical scene recorded using the laparoscope (Supplementary Video). This allowed us to display the three ROIs in real-time onto the pancreas and to chronologically perform, a confocal laser endomicroscopy analysis, local capillary lactate sampling and biopsies in precise correspondence of each ROI.

### 2.4. FLER (Fluorescence-Based Enhanced Reality)

FLER has been extensively described previously [8,9,21]. In a nutshell, the AR-Perfusion™ software version 1 (IRCAD, Strasbourg, France) computes the speed required for the fluorescence signal to reach its maximum, pixel-by-pixel, and the metric used for the current study was the slope of fluorescence speed (FLER slope: ∆intensity/time). Thus, with the same method previously explained elsewhere [9], the dynamic perfusion cartogram of the pancreas was created by averaging fluorescence signals for a 40 s video at a speed of 25 frames per second, and by assigning a color code based on the time required to reach the maximum intensity of each pixel.

However, since the fluorescence signal intensity decreases with distance according to the inverse quadratic law, a calibration reference card providing a constant fluorescence signal (Green Balance™, Diagnostic Green GmbH, Aschheim, Germany) was placed in the surgical field beside the pancreas. The absolute fluorescence intensity signal was normalized by dividing it by the signal provided by the reference card.

### 2.5. Image Warping

Using a previously described algorithm [21], based on the moving least square method [27], the static HSI picture was deformed in order to fit onto the perfusion cartography obtained via FLER, and both images were superimposed onto the live video provided by means of the HD laparoscope. This process was achieved by manually selecting several features visible on both HSI and FLER images. As a result, the software generated the warping automatically. The entire process took around three minutes and the overall quality of the image warping was sufficiently acceptable.

### 2.6. Confocal Laser Endomicroscopy (CLE)

A quantitative histological evaluation was performed using confocal laser microscopy (CLE) (Cellvizio™, Mauna Kea Technology, Paris, France) [22]. CLE provides a real-time virtual image of histology which could be important additional information on the biochemical changes of the pancreatic tissue undergoing ischemia. Immediately after a systemic injection of fluorescein (Fluocyne^TM^, Serb, Paris, France), the pancreatic surface was manually scanned at each ROI and recorded for approximately 45 s. The videos were analyzed using the IC Viewer software™ (version 3.8.5) (Mauna Kea Technologies, France). As previously described in bowel ischemia models [22,23], the software automatically recognizes the vessels as elongated shapes containing contrast and computes the functional capillary density area (FCD-A) index. The FCD-A index is obtained by multiplying the mean capillary diameter by the total vessel length, and the result is then divided by the entire round image area.

During data acquisition of all imaging modalities, in order to minimize bias incidence, environmental lights were turned off and the ventilation was briefly stopped.

### 2.7. Local Capillary Lactate (LCL) Measurement

Immediately after the completion of the optical imaging techniques, biopsies were taken in correspondence with the ROIs and LCL was sampled from the resulting blood. LCL value measurements were performed using a portable analyzer (EDGE^TM^, Apex Biotechnology Corp., Hsinchu, Taiwan). LCL levels robustly correlate with gastrointestinal ischemia, as previously described [8,9,19,21,28].

Systemic lactatemia was measured on blood samples obtained by puncturing the pigs’ snouts, and normalized LCL values were obtained by subtracting the systemic values from LCLs.

### 2.8. Mitochondrial Respiratory Chain Assessment

The assessment of the mitochondrial respiratory chain was performed on the biopsies obtained in correspondence with the ROIs. This provides insights into the energetic status of the tissues by calculating the amount of oxygen that is consumed by the mitochondria. The specimens were placed in a 2 mL water-jacketed oxygraphic cell (Oxygraph-2k^TM^, Oroboros Instruments, Innsbruck, Austria) with a Clark electrode. Mitochondrial activity was measured as previously described [8,9,23,29]. After the determination of basal oxygen consumption (V0), tissue respiration in the presence of a saturating amount of substrate adenosine diphosphate (VADP) was calculated to evaluate complexes I, III and IV of mitochondrial respiration. The respiratory control ratio (RCR = VADP/V0) was computed [30]. The RCR represents the efficiency of coupling between phosphorylation and oxidation. It is the key bioenergetic transduction in the mitochondria. The electron flow passed further through complexes I, III and IV, due to the presence of glutamate (5 mmol/L) and malate (2 mmol/L). For the evaluation of complexes II, III and IV (Vsucci), succinate was injected (25 mmol/L). The addition of rotenone (0.5 mmol/L) allowed the calculation of Vrot, which represents complexes II, III, IV and V activities [31]. For the evaluation of cytochrome c oxidase (complex IV) separately (Vtmpd), N,N,N′,N′-tetramethyl-p-phenylenediamine dihydrochloride (TMPD, 0.5 mmol/L) and ascorbate (0.5 mmol/L) were added as an artificial electron donor to cytochrome c. Finally, tissues were harvested and dried (15 min at 150 °C) and respiration rates were expressed using pmol of oxygen/second/mg of wet weight.

### 2.9. Histology

H&E staining was performed for the biopsy sections of each ROI. The presence of an acute ischemic condition was investigated by a fully trained pathologist. The identity of each ROI was blinded to the pathologist.

### 2.10. Prediction of Local Capillary Lactates from the FLER-Slope and HSI-StO2

The relationship between LCL and the FLER slope and HSI-StO2 respectively followed an exponential trend, which was modeled by exponential regression analysis. This allowed us to generate a prediction algorithm of LCL values from the corresponding HSI-StO2 and FLER slope. In order to validate prediction models, a mean square minimization of the objective function exp (−ax + b) was performed on normalized LCLs using the SciPy, Python library [32]. The prediction functions were obtained from the whole datasets because no significant difference in terms of error prediction could be observed when performing a leave-1 (pig)-out cross-validation. The accuracy of both models, indicated as the absolute difference between the sampled LCLs and the predicted LCL values of both prediction models, was computed on the whole data by the corresponding exponential equation.

### 2.11. Sample Size Calculation

Using the LCL prediction as the primary outcome, a sample size calculation was performed. Based on our previous experiences with bowel ischemia [19,21], we found that HSI-StO2 and LCLs (337 paired values) have a negative correlation coefficient of −0.78. When modeling for sample size calculation using this coefficient (with alpha at 0.05 and a power of 0.9), the required sample size was 13 paired values. In the present study, we had 18 paired values from six pigs, and the correlation coefficient between HSI-StO2 and LCLs in the present study (−0.88) was higher than the one in the previous model (−0.78).

### 2.12. Statistical Analysis

Data are shown as mean ± standard deviation unless indicated otherwise. Statistics were performed using the GraphPad Prism 8 software and Python Scikit-learn library [32]. ANOVA followed by Tukey’s multiple comparison test was used to compare the results among the three zones. Spearman’s rank correlation test was used to investigate variables presenting a non-linear relationship. Exponential regression was also used to investigate predicted LCL values based on HSI-StO2 and the FLER slope. The Wilcoxon test was performed for paired comparison of lactate prediction algorithms, since a non-Gaussian data distribution was observed. A *p*-value < 0.05 was considered statistically significant.

## 3. Results

The endovascular procedures were successful (Figure 1) followed by imaging analyses (Figure 2a). Pancreatic ischemia could be obtained in all pigs (Figure 2b). Additionally, augmented reality (AR) of the overlaid HSI (Figure 2c) and FLER (Figure 2d) images was possible in all cases, and the virtual demarcation line between vital and ischemic zones was visually consistent in both imaging modalities (Supplementary Video). Besides, AR allowed for accurate confocal laser endoscopy (CLE) scanning, local lactate (LCL) sampling and biopsies, in correspondence with each ROI.

HSI

  The mean HSI-StO2 value was 47.3 ± 25.9% and 76.5 ± 6.0% in vital ROI, 48.8 ± 8.1% in transition ROI and 16.5 ± 4.76% in ischemic ROI.

FLER

  The mean FLER slope value was 4.47 ± 4.45 ∆intensity/time and 6.79 ± 4.43 ∆intensity/time in vital ROI, 5.67 ± 4.93 ∆intensity/time in transition ROI and 0.94 ± 0.6 ∆intensity/time in ischemic ROI.

### 3.1. Correlation between HSI-StO2 and the FLER Slope

**Figure 3:**
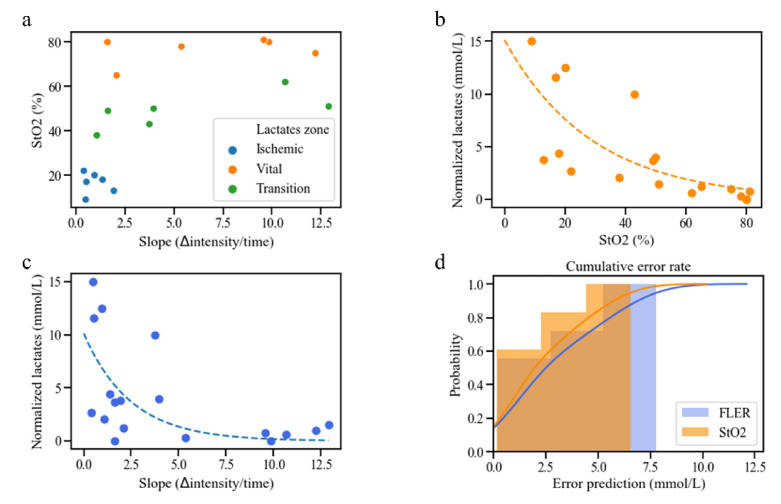
Analyses between HSI-StO2 and the FLER-slope. (**a**) Correlation analysis between HSI-StO2 and the FLER slope in correspondence with the same ROIs showed a R = 0.697 (*p* = 0.013). (**b**) Correlation analysis showed a R = −0.88 (*p* < 0.001) between LCLs and HSI-StO2, (**c**) and a R = −0.608 between LCLs and the FLER slope (*p* = 0.0074). (**d**) HSI-based and FLER-based lactate prediction models had statistically similar predictive abilities (*p* = 0.112). See the following link URL for the explanation of the unit of slope (∆intensity/time). https://www.quora.com/What-is-the-unit-of-a-slope.

The Spearman analysis between HSI-StO2 and the FLER slope in correspondence with the same ROIs provided a R = 0.697, which proved to be statistically significant (*p* = 0.013) (Figure 3a).

### 3.2. Local Capillary Lactate Values

Mean systemic lactate concentration was 1.87 ± 0.869 mmol/L and mean normalized LCL concentration was 4.18 ± 4.74 mmol/L. Mean LCL concentration was 10.2 ± 5.56 mmol/L in the ischemic ROI, which was statistically higher than in the vital ROI (2.42 ± 0.79 mmol/L, *p* = 0.0017) and transition ROI (4.37 ± 1.51 mmol/L, *p* = 0.011). The difference between the vital zone and the transition zone was not statistically significant (*p* = 0.47).

### 3.3. Correlation between HSI, FLER and Local Capillary Lactate

The correlation between LCLs and HSI-StO2 (*p* < 0.001) gave a Spearman’s R = −0.88 **(**Figure 3b), and the correlation between LCLs and the FLER slope (*p* = 0.0074) gave a R = −0.608 (Figure 3c).

### 3.4. Lactate Prediction Models Based on HSI-StO2 and the FLER Slope

The following fitting functions were found for LCL prediction:

1. based on HSI-StO2:
  Predicted LCL = e^−0.0343*HSI StO2+2.72^ + Systemic Lactates
2. based on the FLER slope:
  Predicted LCL = e^−0.403*FLER slope+2.32^ + Systemic Lactates

The lactate prediction model based on HSI-StO2 showed a mean error of 2.36 ± 2.05 mmol/L, with a median error of 1.24 mmol/L, and 95% of the errors occurred for LCLs < 5.98 mmol/L. The mean error of the lactate prediction model based on the FLER slope was 2.93 ± 2.44 mmol/L, with a median error of 1.75 mmol/L, and 95% of the errors occurred for LCLs < 6.88 mmol/L.

The Wilcoxon test between the two prediction models did not show any difference (*p* = 0.112) (Figure 3d).

### 3.5. Mitochondrial Respiratory Chain Assessment

**Figure 4:**
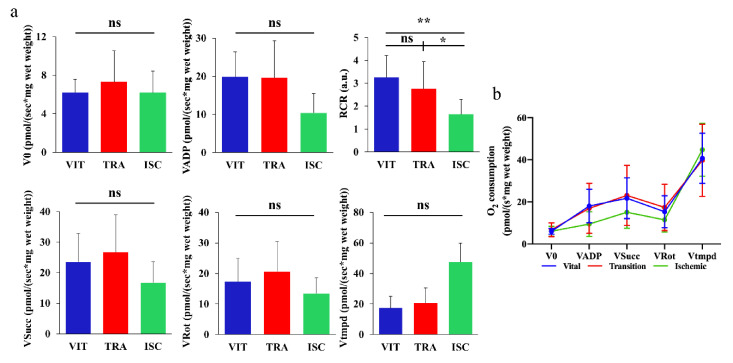
Mitochondrial respiratory test. (**a**) Mean V0, VADP, Vsucc, Vrot, Vtmpd in vital, transition and ischemic zones did not show any statistically significant difference. The mean RCR (VADP/V0) was statistically significantly decreased in the ischemic zone (1.532 ± 0.7295), as compared to the vital zone (2.987 ± 1.048, *p* = 0.0015) and to the transition zone (2.348 ± 1.205, *p* = 0.0465). (**b**) The line charts of three different zones among the five values concerning mitochondrial activity are shown (Mean ± SD) * *p* < 0.05, ** *p* < 0.01. VIT: vital zone, TRA: transition zone, ISC: ischemic zone, ns: not significant.

As shown in Figure 4a, mean V0 and VADP in vital ROI (6.013 ± 1.359 and 18.01 ± 7.97 pmol O2/s/mg wet weight, respectively), transition ROI (6.784 ± 3.272 and 16.94 ± 11.88 pmol O2/s/mg wet weight, respectively) and ischemic ROI (6.064 ± 2.35 and 9.481 ± 5.868 pmol O2/s/mg wet weight, respectively) did not show any statistically significant difference. Nevertheless, RCR (VADP/V0) reflecting mitochondrial coupling was significantly decreased in the ischemic zone (1.532 ± 0.7295 pmol O2/s/mg wet weight), compared to the vital ROI (2.987 ± 1.048 pmol O2/s/mg wet weight, *p* = 0.0015) and to the transition ROI (2.348 ± 1.205pmol O2/s/mg wet weight, *p* = 0.0465). The mean Vsucc/Vrot/Vtmpd in the vital ROI (21.71 ± 9.653/15.33 ± 7.584/40.67 ± 11.9 pmol O2/s/mg wet weight), transition ROI (23.06 ± 14.25/17.38 ± 11.0/39.74 ± 17.14 pmol O2/s/mg wet weight) and ischemic ROI (15.1 ± 7.629/11.49 ± 5.811/44.7 ± 12.56 pmol O2/s/mg of wet weight) did not present any statistical differences. The line charts concerning mitochondrial activity are shown in Figure 4b.

### 3.6. Confocal Laser Endomicroscopy (CLE) and Histological Findings

**Figure 5:**
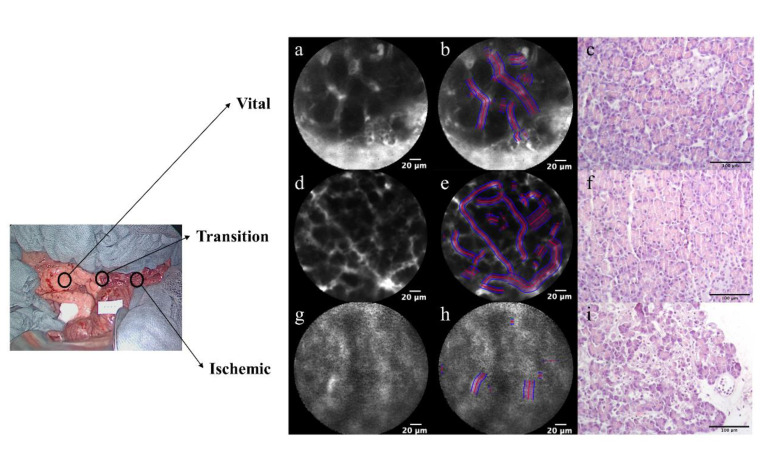
Confocal laser endomicroscopy (CLE) with functional capillary density area (FCD-A) index analysis and histology. (**a**,**d**,**g**) The findings of CLE are shown in vital, transition and ischemic zones. By increasing the permeability of vascular and pancreatic acinar cells, a pooling phenomenon of the fluorescein on the pancreatic surface was gradually strengthened from vital to ischemic zones. (**b**,**e**,**h**) The FCD-A index is shown in the still images of CLE. The FCD-A index was significantly more decreased in the ischemic zone (**h**: 0.122 ± 0.03) than in the vital zone (**b**: 0.26 ± 0.041, *p* < 0.0001) and transition zone (**e**: 0.247 ± 0.02, *p* < 0.0001). (**c**,**f**,**e**) Histological findings are shown (20× objective lens) (**c**) in vital, (**f**) transition and (**i**) ischemic zones.

CLE videos were successfully recorded at each ROI of the pancreatic surface in all the pigs (Figure 5a,d,g). The mean functional capillary density area (FCD-A) index was significantly more decreased in the ischemic ROI (0.122 ± 0.03) than in the vital ROI (0.26 ± 0.041, *p* < 0.0001) and the transition ROI (0.247 ± 0.02, *p* < 0.0001) (Figure 5b,e,h). Although the mean FCD-A index was slightly decreased in the transition zone, the difference was not statistically significant compared to the vital zone (*p* = 0.853). Regarding histological findings, it was possible to see normal pancreatic acinar cells in the vital zone and no ischemic changes were observed (Figure 5c). In the transition zone, the architecture of pancreatic acinar cells appeared to be preserved. However, some pale areas were observed, which seemed to indicate the focal suffering of pancreatic acinar cells (Figure 5f). In the ischemic zone, an irregular area was diffusely observed, which represented necrosis of acinar cells with apoptotic figures, edema and granulocytic inflammatory infiltration. At higher magnification, neutrophil granulocytes were adherent to the vessel wall, which is characteristic of reactive phenomena with neutrophil migration to inflammation areas in the adjacent blood vessels of pancreatic acinar cells. This aspect could reflect initial ischemia (Figure 5i).

## 4. Discussion

Preventing pancreatic fistulas has been a central discussion topic among pancreatic surgeons over the last years and a number of clinical or preclinical studies aimed at understanding the conditions implicated in PF formation have been performed [33]. Some authors have previously affirmed that poor perfusion of the pancreatic resection surface may play an essential role in PF development [6,34]. This theory is supported by the fact that the vascular supply of the pancreas is highly variable [35] and a poor vascular area in correspondence with the pancreatic neck has been well-described during previous anatomical studies [35,36]. In this view, since pancreatic head resections imply that the resection surface coincides with the neck, this operation is likely to generate poorly perfused pancreatic resection surfaces, which could concur with postoperative PF development. Accordingly, Strasberg et al. noticed that the PF rate dropped consistently if meticulous care was taken to perform pancreatic anastomoses exclusively using a well-perfused resection surface [6]. However, there are very few studies that further explored the association between poor perfusion and PF formation in a reproducible manner. Initially, we aimed at achieving selective pancreatic head ischemia, in order to simulate the vascular situation present during surgical pancreatic head resection. However, since the porcine pancreatic head is richly vascularized through an interconnected capillary network, complete pancreatic head embolization failed in a set of preliminary experiments. As a result, we decided to embolize the arteries heading to the pancreatic tail, which resulted in segmental pancreatic tail ischemia in all the animals involved in the study. Therefore, in our pilot study, the main pancreatic branch of the splenic artery (equivalent to the dorsal pancreatic artery in humans [37]) was selectively embolized. This simulates the arterial ligation in a distal pancreatectomy, which would be the necessary step prior to parenchymal transection. The choice to assess pancreatic perfusion two hours after vascular embolization was made in order to simulate the workflow of a surgical procedure. In fact, during pancreatic head resection, the division of the gastroduodenal artery takes place approximately two hours prior to pancreatic parenchyma transection.

Even though the ischemic area was visible to the naked eye a few minutes following the endovascular vessel occlusion, the optical imaging modalities we adopted, namely FLER and HSI, allowed us to precisely quantify and visualize real-time the perfusion of the entire pancreas. If our findings result in reproducible during human pancreatectomy, we could theoretically exactly determine the ideal resection line in accordance with perfusion status displayed by the two imaging modalities, and this might prevent PF. Currently, a human trial is ongoing, and this will show the relevance of our results in the clinical practice. In fact, we chose to employ optical imaging technologies, due to their minimal impact on the surgical workflow. This enables theoretically immediate clinical translation, which is corroborated, in the case of HSI and fluorescence imaging, by the high spatial colocalization offered by the enhanced reality implementation (HYPER and FLER). On the contrary, CLE, being a probe-based technology, provides only a microscopic punctual spatial information, limiting de facto its utilization in the daily clinical practice.

Nevertheless, FLER, HYPER and CLE provide surrogate blood flow parameters and do not measure the red blood cells velocity (perfusion) itself. However, the accuracy of those three imaging modalities to precisely quantify ischemia within the gastrointestinal tract has been previously validated with in vivo experiments using robust biomarkers [8,9,19,20,21,22,23,24,25]. On the other hand, another optical imaging modality, the laser speckle contrast imaging (LSCI), is able to detect the real-time perfusion. Despite some promising results within gastrointestinal surgery [38,39,40,41,42], this technology suffers from unsolved issues, which limit its potential intraoperative utilization [43]. Firstly, respiration and heartbeat easily cause large measurements’ artifacts. Secondly, this technique is still bound to qualitative assessment, therefore it has not been validated yet using strong biological markers. Thirdly, interpatient comparability of LSCI is rather problematic, since a highly controlled environment containing a no-flow region is necessary, and this is troublesome to reproduce in a clinical setting.

The results of the present study showed that both HSI and FLER could provide real-time perfusion information, and both metrics, HSI-StO2 and the FLER slope, were statistically correlated. Additionally, both parameters had statistically significant exponential relationships with LCL values, and this allowed us to extrapolate two LCL prediction models. Interestingly, HSI-StO2 showed a stronger correlation with LCL than FLER-Slope. The reason could be that the duration of ischemia in the present study was only 2 h and possibly within this time lapse only microscopic intracellular metabolic changes occur. HSI seems to have better sensitivity for detecting the microscopic changes of early ischemia than FLER, as previously observed in a small bowel early ischemia model [21].

Our group has established the augmented reality of HSI and FLER, which can be superimposed onto the real-time video of the surgical scene. The accuracy of FLER and HYPER had been previously widely validated [8,9,19,21]. However, this is the first time that both technologies are used to intraoperatively assess pancreatic ischemia. In the present study, both technologies correlated with ischemia biomarkers. Importantly, HSI and FLER might be influenced by external factors, such as environmental light and motion. For this reason, during data acquisition, lights were turned off and ventilation was paused for a few seconds. Both precautions are feasible during surgery and do not disrupt consistently the operational workflow. Additionally, a clinical trial using FLER during laparoscopic colorectal surgeries has been recently published [44] and it was possible to prove the feasibility and functionality of this technology in daily practice. A clinical trial using HYPER is currently ongoing, but since HSI is a contrast-free technology, the acquisition time is even shorter than FA and the method to obtain enhanced reality is similar to FLER, a smoother clinical translation might be expected.

CLE is a powerful endoscopic-assisted optical imaging modality which allows obtaining a histopathological evaluation of the targeted tissue [45]. This technology demonstrated high sensitivity and specificity in the detection of dysplasia in Barrett’s esophagus, gastric neoplasm, colorectal cancers in inflammatory bowel disease, pancreatobiliary strictures and pancreatic cysts [45]. However, the quality of the assessment is highly operator dependent and the result could be subjective without any quantitative assessment. Our group previously and successfully assessed the precision of the FCD-A index, a quantitative index of perfusion, in detecting bowel ischemia [22,23]. The FCD-A index is based on the amount of fluorescein signal detection, computed by the vessel detection tool included in the software. In theory, it might be well correlated to the findings of FLER, which captures the fluorescent emission in the capillary system. In the early ischemic phase, leaks of fluorescein were theoretically observed by increasing the permeability of vascular and pancreatic acinar cells. A 2-h ischemic injury led to a pooling phenomenon homogeneously distributed on the pancreatic surface. As a result, we found a statistically significant decrease in the mean FCD-A index in the ischemic zone, when compared to the other zones. The abolishment of the capillary system in the ischemic zone was also consistently verified in the histology.

The mitochondrial activity investigation was performed to assess the cellular energy environment. Oxidative phosphorylation produces energy for the cells with a series of reduction/oxidation reactions in mitochondria. Electrons are transferred sequentially from donors (NADH or QH2) to the most electronegative acceptor, namely oxygen. In this electron transport chain, an electrochemical gradient across the mitochondrial membrane is produced by actively pumping H+ protons from the matrix to the intermembrane cavity. This electrochemical gradient is used to synthesize adenosine triphosphate which is the cellular currency of energy. There are three major enzymatic proton pumps (i.e., complex I, III and IV). In our 2-h ischemic model, we did not find any statistically significant difference in O2 consumption in any ROI. The reason why the mitochondrial impairment was generally limited might be explained by (i) short ischemic time (2 h), (ii) lack of ischemia-reperfusion injury and (iii) collateral arterial flow from the superior mesenteric artery in pig pancreas [37]. Nevertheless, a deficit of energetic coupling (i.e., RCR) was observed in the ischemic zone. This suggested an early phenomenon of mitochondrial impairment that precedes defects in the electron transport chain.

## 5. Conclusions

In conclusion, both FLER and HSI allow to precisely quantify and visualize real-time perfusion of the pancreas in this porcine model of pancreatic ischemia. HSI-based and FLER-based lactate prediction models had statistically similar predictive abilities in the present setting (*p* = 0.112). The imaging analyses showing the pancreatic ischemic condition were verified by means of the cellular parameter, as the FCD-A index was significantly decreased in the ischemic zone than in the vital zone (*p* < 0.0001) and the transition zone (*p* < 0.0001). In addition, the imaging analyses were also verified by means of physiological findings, as RCR (VADP/V0) was significantly decreased in the ischemic zone than in the vital ROI (*p* = 0.0015) and in the transition zone (*p* = 0.0465). Histological findings have also confirmed the same findings of the imaging analyses. Currently, the capability of HSI and FLER is being investigated for human cases, and if its utility in preventing PF is demonstrated, it might have a relevant impact on the outcome of oncological patients undergoing pancreatic surgery.

## Data Availability

The datasets generated or analyzed during the current study are included in this published article.

## Supplementary Materials

The following are available online at https://www.mdpi.com/2075-4418/11/1/93/s1, Supplementary video: Augmented reality of the overlaid HSI and FLER images. The warped image of the HSI and the FLER image generated by the software were overlayed onto the live video of the surgical scene recorded using the laparoscopic camera. The quality of the image warping was generally acceptable in visual perception as the images were well fitted to pancreatic structures. The virtual demarcation line between vital and ischemic zones was visually consistent in both imaging modalities. This allowed for accurate confocal laser endoscopy scanning, local lactate sampling and biopsies in correspondence with each ROI.
